# Loss of Endothelial Glycocalyx Hyaluronan Impairs Endothelial Stability and Adaptive Vascular Remodeling after Arterial Ischemia

**DOI:** 10.3390/cells9040824

**Published:** 2020-03-29

**Authors:** Gangqi Wang, Margreet R. de Vries, Wendy M. P. J. Sol, Annemarie M. van Oeveren-Rietdijk, Hetty C. de Boer, Anton Jan van Zonneveld, Paul H. A. Quax, Ton J. Rabelink, Bernard M. van den Berg

**Affiliations:** 1 The Einthoven laboratory for Vascular and Regenerative Medicine, Department of Internal Medicine, Division of Nephrology, Leiden University Medical Center, 2333 ZA Leiden, The Netherlands; g.wang@lumc.nl (G.W.); W.M.P.J.Sol@lumc.nl (W.M.P.J.S.); A.M.van_Oeveren-Rietdijk@lumc.nl (A.M.v.O.-R.); H.C.de_Boer@lumc.nl (H.C.d.B.); A.J.van_Zonneveld@lumc.nl (A.J.v.Z.); A.J.Rabelink@lumc.nl (T.J.R.); 2 The Einthoven laboratory for Vascular and Regenerative Medicine, Department of Surgery, Leiden University Medical Center, 2333 ZA Leiden, the Netherlands; M.R.de_Vries@lumc.nl (M.R.d.V.); P.H.A.Quax@lumc.nl (P.H.A.Q.)

**Keywords:** hyaluronan, endothelial glycocalyx, angiogenesis, diabetes

## Abstract

We recently reported that loss of hyaluronan (HA) from the endothelial glycocalyx leads to loss of vessel stability in specific microcirculatory vascular beds. Here we hypothesized that such derangements in the glycocalyx may also impair the adaptive response to vascular ischemia. Endothelial specific conditional hyaluronan synthase 2-KO (*Has2*-cKO) mice revealed reduced endothelial HA expression and lower hindlimb perfusion at baseline compared to control mice. After a single ligation of the common femoral artery in these mice, we observed dysregulated angiogenesis in the gastrocnemius muscle which did not restore capillary perfusion. Mechanistically, decreased endothelial binding of the pericyte-derived molecule angiopoietin1 (Ang1) could be observed in the *Has2*-cKO mouse. In vitro angiogenesis assays with an endothelial cell-pericyte coculture confirmed such disturbed Ang1-TIE2 signaling resulting in excessive angiogenesis upon loss of HA. These data could be of relevance to diabetes patients, where we confirm loss of endothelial HA in the microcirculation of muscle tissue, indicating that this may contribute to the known disturbed adaptation to ischemia in these patients. In summary, loss of endothelial HA results in impaired microvascular perfusion and endothelial stability in ischemic gastrocnemius muscle. Endothelial HA is a potential target to improve angiogenic therapy in diabetic patients with critical limb ischemia.

## 1. Introduction

During peripheral artery disease, occlusion of the arteries can trigger a series of compensatory events, such as arteriogenesis and angiogenesis, to restore perfusion in the ischemic tissue [1,2]. One therapeutic strategy that has been explored in this condition is to enhance angiogenesis through administration of growth factors and vasculogenic cells [1]. Although some success of angiogenic therapy has been reported in young healthy animal models after acute artery ligation, these therapies failed to demonstrate clear-cut evidence of therapeutic efficacy in patient groups, such as diabetes [1,3,4].

The endothelial glycocalyx is a negative charged gel-like structure coating the surface of endothelial cells (ECs), which is critically involved in vascular integrity and homeostasis, such as vascular permeability, mechanotransduction, inflammation and coagulation [5,6]. For vascular homeostasis the selective binding capacity of growth factors and chemokines to endothelial glycocalyx is essential [7,8,9,10]. In pathological conditions, like inflammation, hyperglycemia, and atherosclerosis, the endothelial glycocalyx is degraded by enzymes such as heparanase and hyaluronidases [11,12,13]. Such impaired endothelial glycocalyx results in endothelial dysfunction and is associated with a less efficient and defective microvascular perfusion [14,15].

Hyaluronan (HA) is one of the main structural constituents of the endothelial glycocalyx. It is synthesized by the synthases HAS1, HAS2, or HAS3 at the inner face of the plasma membrane and is secreted into the extracellular space. Mice that are deficient for *Has2* die early in gestation due to major defects in cardiovascular development, suggesting that HA may function as a molecular platform for vascular signaling [16]. Shedding of endothelial HA was observed in both type 1 and type 2 diabetic patients [17,18], and is considered a causative factor of diabetic vascular and renal complications. Removal of endothelial HA with the HAS inhibitor 4-methylesculetin (4ME) greatly impaired the collateral artery remodeling process, in a femoral occlusion model, due to perturbed mechanotransduction and in turn lack of activation of the smooth muscle cells, necessary for vascular remodeling [19]. In particular, the critical involvement of HA microvascular complications such as in diabetes has been reviewed recently [20]. However, its role in microvascular perfusion and the process of angiogenesis is still not clear.

In the present study we investigate the role of endothelial HA in restoring the microvascular perfusion in response to femoral arterial obstruction with regard to the angiogenic potential in the gastrocnemius muscle. To explore the potential clinical significance of endothelial HA loss, we determined HA expression in ischemic muscle biopsy tissue of diabetic patients with critical limb ischemia.

## 2. Materials and Methods

### 2.1. Animal Experiments

All experiments were carried out in conditional endothelial specific has2 knockout (*Has2*-cKO) mice or control mice in compliance with the Dutch government, the Directive 2010/63/EU of the European Parliament, and approved by the ethical committee on animal care and experimentation of the Leiden University Medical Center (permit no. 14-150). Presence of introduced genes from ear pinched DNA samples is performed on all animals during breeding and for confirmation of has2 gene mutation (exon 2 deletion) after tamoxifen on *Has2*-cKO mice at the end of the experiment using PCR and primers, as described earlier [10]. Only *Has2*-cKO mice with a confirmed mutation within their has2 gene were examined further. In short, eight-week-old male *Has2*-cKO and control mice received intra-peritoneal 2 mg/0.2 mL tamoxifen for 5 consecutive days, resulting in a about 80% tamoxifen induced gene re-editing efficiency in calf muscle tissue (Supplementary Figure S1A). At 4 weeks after tamoxifen, a single ligation surgery or a double ligation surgery was performed in *Has2*-cKO and control mice, followed with Laser Doppler Perfusion Imaging (LDPI) perfusion and body weight measurement for 14 days. Blood was taken at day one, before ligation, and after 14 days (sacrifice). Under Isoflurane anesthesia, mice were killed by heart puncture for blood collection and perfusion fixation. Both adductor muscle and gastrocnemius muscles were collected and embedded in paraffin. 

### 2.2. Hindlimb Ligation Surgery and Laser Doppler Perfusion Imaging (LDPI) 

The details of double and single ligation surgery were described in the previous study [21,22]. In brief, for a double ligation model, both common iliac artery and femoral artery were electrocoagulated, and for single ligation model, the left common femoral artery proximal to the bifurcation of the popliteal and saphenous artery was electrocoagulated [22]. Mice were anesthetized before surgery and the anesthesia was antagonized after surgery as descripted in Supplemental Material. Before LDPI mice were anesthetized with an intra-peritoneal injection of midazolam and medetomidine and mice were kept in a double-glassed jar filled with a 37°C water mantle during the whole measurement. Before and after ligation, perfusion was measured using Laser Doppler Perfusion Imaging (Moor Instruments). The perfusion values were analyzed using Moor LDI V5.3 software. The perfusion rate measured, normalized by capillary density, was used to calculate capillary perfusion capacity.

### 2.3. Human Tissue Samples

Human tissue samples from patients with critical limb ischemia and type 2 diabetes were collected at the Leiden University Medical Center. This study was conducted according to the Declaration of Helsinki. Sample collection was approved by the Medical Ethics Committee of the Leiden University Medical Center (Protocol No. P12.265) and written informed consent was obtained from the participants.

Inclusion criteria were a minimum age of 18 years and lower limb amputation, excluding ankle, foot, or toe amputations. The exclusion criteria were suspected or confirmed malignancy and inability to give informed consent. Tissue samples were obtained directly after lower limb amputation, snap-frozen, and stored at −80 °C.

### 2.4. Histology 

Deparaffinized muscle sections (4 µm thick) were rinsed in Millipore water and stained with Mayer’s Haematoxylin (109249, Merck, Darmstadt, Germany) for 1 min. Slides were rinsed in tap water and kept on an orbital shaker for 10 min for further rinsing with milliQ water. Next, slides were incubated with an ethanol-based eosin (E6003, Sigma-Aldrich, Zwijndrecht, the Netherlands) solution for 2 min. Slides were dehydrated in absolute ethanol and xylene and mounted.

### 2.5. Immunohistochemistry

Deparaffinized muscle sections (4 µm thick) were washed in PBS and antigen retrieval was performed using antigen retrieval buffer (Dako, Santa Clara, CA, USA) in an autoclave. Slides were blocked in Serum-Free Protein Block buffer (Dako) for 1 h at room temperature. Primary Goat anti-mouse Ang1 (AF923, R&D Systems, Minneapolis, MN, USA), rabbit anti laminin (Z0097, Dako), rat anti-mouse PDGFβ (ab51876, Abcam, Cambridge, UK), HA binding peptide Neurocan-dsRed [10] (200 µg/mL), or lectin from *Bandeiraea simplicifolia* isolectin B4 (BS-1-TRITC, L5264, Sigma or BS-1-BIOTIN, L2140, Sigma, Zwijndrecht, the Netherlands) were incubated overnight at 4°C in blocking buffer, followed by an appropriate secondary antibodies for 1 h at room temperature. Human diabetic muscle sections (4 µm thick) of critical limb ischemia biopsies treated as above and incubated overnight with Ncan-dsRed (200 µg/mL) and Rabbit anti-Human CD31 antibody (ab28364, Abcam) at 4 °C, followed by an appropriate secondary antibody for 1 h in blocking buffer, washed with PBS. Tissue slides were embedded in Prolong^TM^ gold antifade mountant with DAPI (P36931, ThermoFisher, Bleiswijk, the Netherlands) and recorded using a 3D Histech Pannoramic MIDI Scanner (Sysmex, Budapest, Hungary). Quantification was performed using the public domain NIH ImageJ software (FIJI version 1.49m; http://rsb.info.nih.gov/ij). 

### 2.6. Viral Transduction

The hyaluronan synthase 2-short-hairpin RNA lentivirus (pLV-*CMV-IE.HAS2shRNA*) was created through transfecting Hek293 cells as described in Supplemental Materials. Primary human glomerular-derived microvascular endothelial cells (hMVECs) or primary human umbilical vein endothelial cells (HUVECs) were cultured to 60–80% confluency in T75 flasks (Greiner bio-one, Alphen a/d Rijn, the Netherlands) in EGM2 (Lonza) medium and transduced with pLV-CMV-IE.HAS2shRNA or mock (pLV-CMV-IE) in combination with 8 µg/mL polybrene, incubated overnight at 37 °C and 5% CO_2_. After medium refreshment, experimental assays were started.

### 2.7. Angiogenesis Plexus Assay

Kidney mesenchymal stromal cells (kMSCs, 2 × 10^4^ cells) and hMVECs (1 × 10^4^ cells) were mixed and seeded in 1% gelatin coated 96 well plate, cultured in EC-SFM medium (Gibco, Zwijndrecht, the Netherlands) with 1% platelet poor plasma derived serum, 30 ng/mL vascular endothelial growth factor 165 (VEGF-165) (R&D, Minneapolis, MN, USA), and 20 ng/ mL fibroblast growth factor-basic (bFGF, Miltenyi, Bergisch Gladbach, Germany). Cells were pelleted by centrifugation for 30 s at 300× *g*. The cells were cultured for 7 days until vascular tubes formed. Next, cells were fixed with 4% PFA and 0.2% Triton-X100 in PBS for 10 min at room temperature, washed with PBS, and blocked for 1 h at room temperature in 5% BSA in PBS. Cells were incubated overnight at 4 °C with primary mouse anti-human CD31 (555445, BD Biosciences, San Diego, CA, USA) and alpha-SMA (C6198, Sigma, Zwijndrecht, the Netherlands), followed by an appropriate secondary antibodies and Hoechst 33528 for 1 h, all in blocking buffer. Cells were examined using a LEICA TCS SP8 X WLL (Leica, Rijswijk, The Netherlands) and a 60× objective (HC PL APO CS2 40x/1.30 OIL, Leica). Sequential 16-bit confocal images (xyz dimensions, 0.142 × 0.142 × 0.3 μm) were recorded using LAS-X Image software (Leica) and analyzed with ImageJ. Both vascular area and vascular branches were quantified.

### 2.8. Enodthelial cell (EC)/pericyte Coculture Assay

Kidney mesenchymal stromal cells (kMSCs, 2 × 10^4^ cells) were seeded in 1% gelatin coated 96 well plate. After 4 h when all the cells adhered to the plate, HUVECs (2 × 10^4^ cells) were added on top of the kMSCs, cultured in EGM2 (Lonza, Basel, Switzerland) medium. The cells were cultured for 2 days until a confluent endothelial monolayer formed. Next, cells were fixed with 4% PFA and 0.2% Triton-X100 in PBS for 10 min at room temperature, washed with PBS, and blocked for 1 h at room temperature in 3% normal goat serum and 2% BSA in PBS. Primary monoclonal Mouse Anti-Human VE cadherin (CD144, 55-7H1, BD Biosciences, San Diego, CA, USA) and phalloidin-TRITC (P1951, Sigma, Zwijndrecht, the Netherlands) were incubated overnight at 4 °C, followed by an appropriate secondary antibody and Hoechst 33528 for 1 h, all in blocking buffer. Cells were examined using a LEICA TCS SP8 X WLL (Leica, Rijswijk, The Netherlands) and a 60x objective (HC PL APO CS2 40x/1.30 OIL, Leica). Sequential 16-bit confocal images (xyz dimensions, 0.142 × 0.142 × 0.3 μm) were recorded using LAS-X Image software (Leica) and analyzed with ImageJ. The stable linear adherence junctions were quantified as ratio over total junction length [23,24].

### 2.9. Real-time PCR and Immunoblotting

Real-time PCR and western blots were performed from samples of HUVECs/kMSCs coculture as described in Supplemental Materials. 

### 2.10. Statistical Analysis 

Data are presented as mean ± SD, unless indicated otherwise. For all experiments, 3–5 biological replicates were performed. All data were performed with Shapiro-Wilk test and Levene’s test to evaluate the normality and variances firstly. For the normally distributed and equal variance data, the differences between two groups were assessed by paired 2-tailed Student’s t test, and between multiple groups were assessed by one-way ANOVA followed by Tukey test. If the data were not normally distributed or not of equal variance, the Mann-Whitney *U* test was performed. *p* values < 0.05 were considered statistically significant.

## 3. Results

### 3.1. Chronic Loss of Endothelial Hyaluronan (HA) Results in Reduced Hindlimb Perfusion

**Figure 1:**
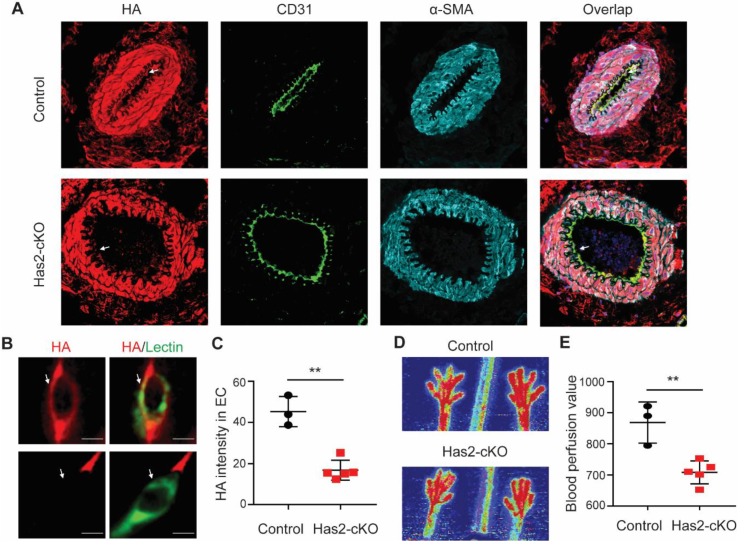
Loss of hyaluronan in endothelial cells (ECs) results in impaired perfusion in hindlimb. (**A**) Representative images of arteries and (**B**) small capillaries in gastrocnemius muscle of endothelial specific conditional hyaluronan synthase 2 KO (Has2-cKO) mice (*n* = 5) and control mice (*n* = 3), stained for the luminal glycocalyx with the hyaluronan (HA) binding probe Ncan-dsRed (red), α-CD31 and lectin from *Bandeiraea simplicifolia* (Green, BS1-lectin) for endothelial localization, α-SMA for smooth muscle cells. (**C**) Quantification of HA intensity colocalized with lectin in ECs. (**D**) Representative images and (**E**) quantification of paw perfusion by laser Doppler perfusion imaging (LDPI) before ligation shows a significantly lower perfusion in the hindlimb of HAS2-cKO. Values are given as mean ± SD. *p < 0.05, **p < 0.01.

At week 8 of age, *has2* gene expression knock-down was induced with tamoxifen and after 4 weeks experimental ligations were applied. Before ligation, endothelial HA expression was significantly reduced in *Has2*-cKO mice as shown in the arteries of the gastrocnemius muscle and luminal site of the capillary endothelial cells (Figure 1A–C) while HA presence in smooth muscle cells was not affected (Figure 1A and Supplementary Figure S1B). As determined by LDPI, overall hindlimb blood perfusion before ligation was significantly reduced upon loss of endothelial HA (Figure 1D,E).

### 3.2. Loss of Endothelial HA Leads to Muscle Damage and Excessive Angiogenesis after Femoral Artery Ligation

**Figure 2:**
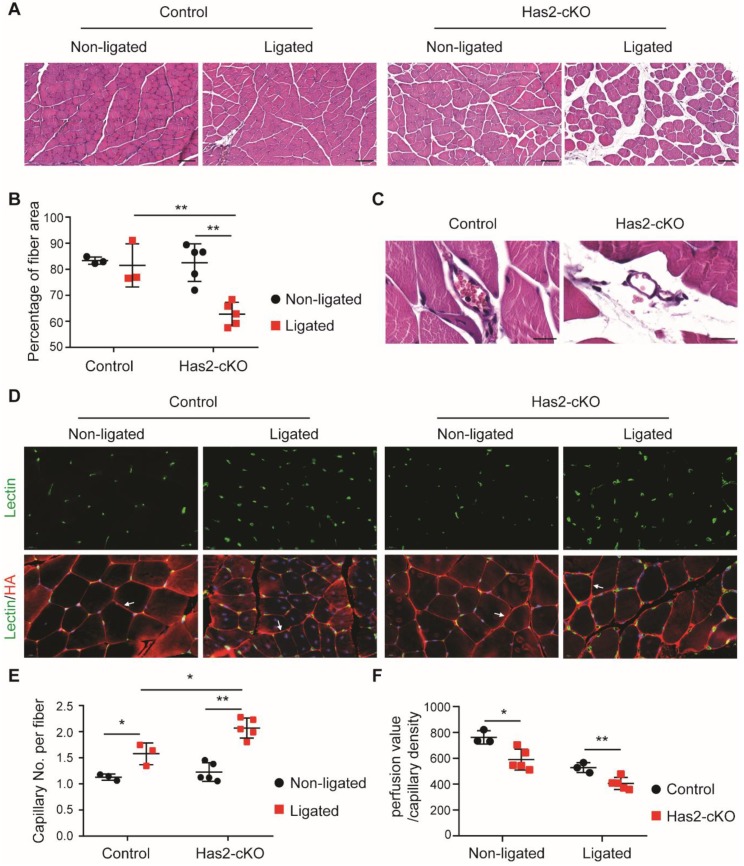
Loss of hyaluronan in ECs leads to muscle damage and excessive angiogenesis after femoral artery ligation. (**A**) Representative images of H&E staining and (**B**) quantification of myocyte area in ligated and non-ligated adductor muscle of Has2-cKO (*n* = 5) and control (*n* = 3) mice (scale bar = 100 µm). (**C**) Representative images of H&E staining show leaky vessels in Has2-cKO mice (*n* = 5) after single femoral artery ligation (scale bar = 20 µm). (**D**) Representative images of BS1-lectin staining for ECs localization (green) and HA staining with the HA binding probe Ncan-dsRed (red). HA staining is used for the myofibroblasts visualization and are found to be surrounded by HA staining as for example depicted by the white arrows. (**E**) quantification of capillary densities in ligated and non-ligated gastrocnemius muscle of Has2-cKO (*n* = 5) and control (*n* = 3) mice. The capillary density is defined as capillary number per myofibroblast. (**F**) quantification of capillary perfusion capacity, calculated from measured LDPI perfusion value, normalized by capillary density in Has2-cKO (*n* = 5) and control (*n* = 3) mice after fully recovery. Values are given as mean ± SD. * *p* < 0.05, ** *p* < 0.01.

To test the effect of reduced endothelial HA in microvascular reperfusion we performed a single ligation surgery on the common femoral artery since, unexpectedly, double ligation surgery such as performed in our previous study [19], resulted in loss of animals within the recovery period (Supplementary Figure S1C). After ligation, initial overall perfusion fully recovered in 10 days in control and *Has2*-cKO mice. However, the perfusion ratio in *Has2*-cKO started to decline again at day 14 (Supplementary Figure S1D,F). Within the adductor muscle tissue, leaky vessels defined by extravasated red blood cells and increased tissue edema between muscle fibers could be observed in *Has2*-cKO compared to control mice (Figure 2A–C). While in the ischemic prone gastrocnemius muscle a significantly excessive capillary density increase was accompanied by increased presence of adipocytes (Figure 2D,E and Supplementary Figure S2). This increased angiogenic response, however, did not result in restoration of blood perfusion (Figure 2F).

### 3.3. Loss of Endothelial HA Reduces Endothelial Angiopoietin 1 Presence

**Figure 3:**
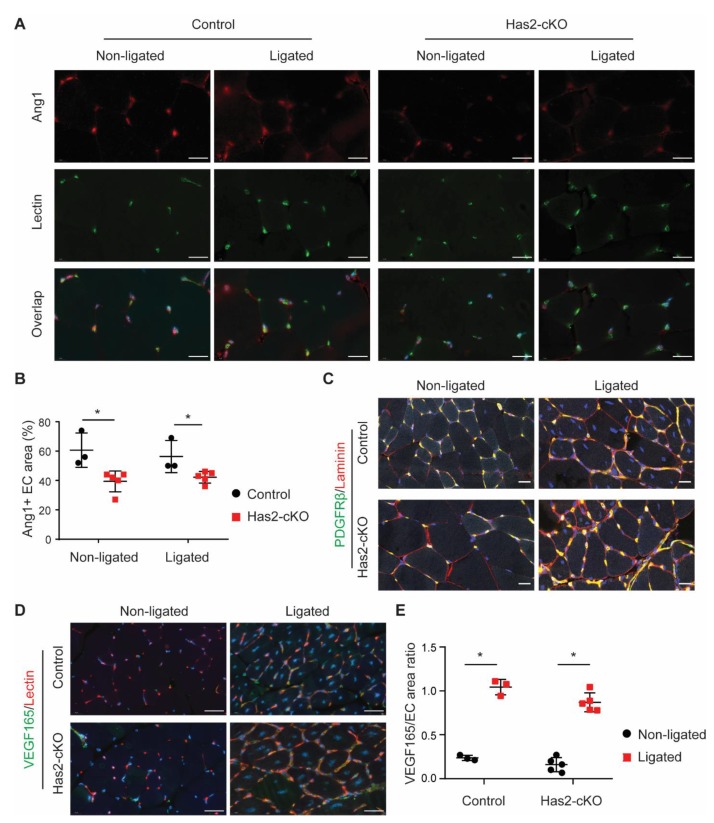
Loss of hyaluronan in ECs decreases angiopoietin 1 presence in ECs. (**A**) Representative images of angiopoietin 1 (Ang1, top) staining and BS1-lectin staining (middle) for ECs localization (scale bar = 20 µm) and (**B**) quantification of endothelial associated Ang1 in ligated and non-ligated gastrocnemius muscle of Has2-cKO (*n* = 5) and control (*n* = 3) mice. (**C**) Representative overlay images of platelet-derived growth factor receptor beta (PDGFRβ, green), laminin (red) and co-staining (yellow) in ligated and non-ligated gastrocnemius muscle of Has2-cKO (*n* = 5) and control (*n* = 3) mice (scale bar = 20 µm). (**D**) Representative overlay images of vascular endothelial growth factor 165 (VEGF165, green), BS1-lectin (red) and (**E**) quantification of VEGF165 and EC area ratio in ligated and non-ligated gastrocnemius muscle of Has2-cKO (*n* = 5) and control (*n* = 3) mice (scale bar = 20 µm). Values are given as mean ± SD. * *p* < 0.05.

Since we found increased angiogenesis accompanied by impaired microvascular perfusion, we tested whether EC destabilization could play a role. For this, the gastrocnemius muscle tissue was stained for vascular stabilization factor angiopoietin 1 (Ang1), pericyte marker platelet-derived growth factor receptor β (PDGFRβ) and basal membrane constituent laminin (Figure 3A–C). Although the capillaries were still covered with pericytes and aligned by a laminin layer, we found a significant reduction in the presence of endothelial bound Ang1 (Figure 3A,B). As expected, the VEGF165 increased in the ischemic gastrocnemius muscles of both control and *Has2*-cKO mice after ligation, which showed no differences between control and *Has2*-cKO mice (Figure 3D,E). These data suggest that the increased angiogenesis in *Has2*-cKO mice after ligation may result from loss of Ang1 presence.

### 3.4. Loss of Endothelial HA Increases Angiogenesis and Endothelial Destabilization In Vitro

**Figure 4:**
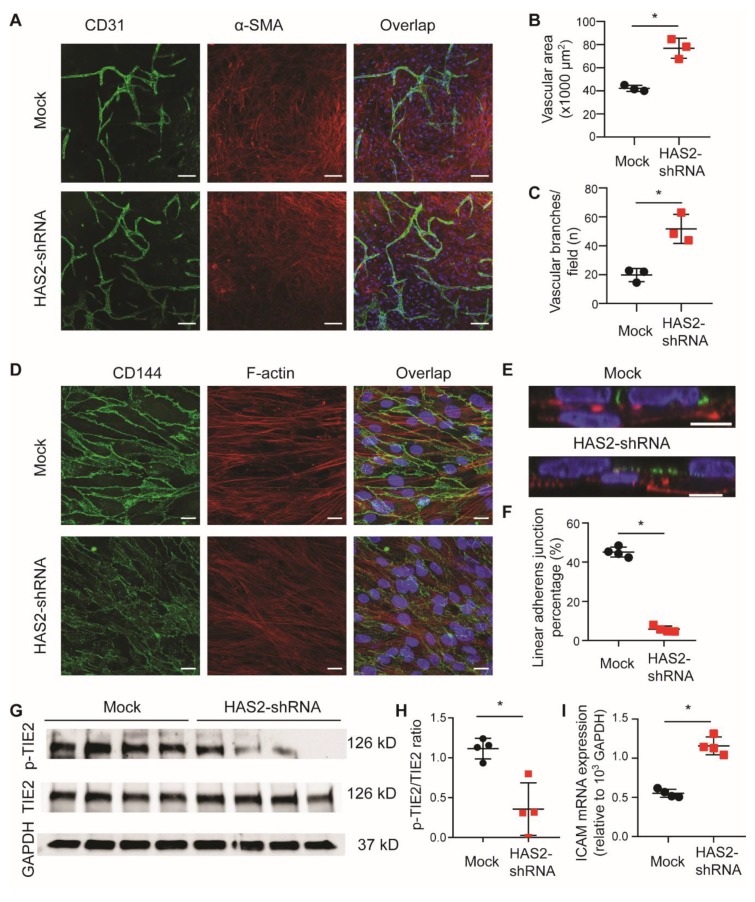
Loss of hyaluronan in ECs increases angiogenesis and endothelial destabilization. To test the function of HA in vitro, a short hairpin RNA (*HAS2*-shRNA) was used to silence HAS2 expression in ECs. (**A**) Representative images of EC-pericyte coculture angiogenesis plexus assay stained with CD31 (green) and α-SMA (red) (scale bar = 100 µm). Quantification of both (**B**) vascular area and (**C**) vascular branches show increased angiogenesis with silencing of HAS2 (*n* = 3). (**D**) Representative images of EC-pericyte coculture (*n* = 4, scale bar = 20 µm) and (**E**) its side view stained for VE-Cadherin/CD144 (green) and F-actin (red), which ECs (VE-cadherin positive) stay on top of pericytes (scale bar = 10 µm). (**F**) Quantification of linear adherens junctions shows endothelial destabilization with silencing of HAS2 (*n* = 4). (**G**) Images and (**H**) quantification of western blot for p-Tie2, Tie2 and GAPDH show a reduced Tie2 activation with silencing of Has2 (*n* = 4) compared to control (*n* = 4). (**I**) An increased ICAM mRNA expression is shown with silencing of Has2 (*n* = 4), indicates ECs activation after loss of HA. Values are given as mean ± SD. * *p* < 0.05.

To further determine the role of HA in the endothelia and pericyte interactions, we used a short hairpin RNA (HAS2-shRNA) to silence *HAS2* expression in ECs (Supplementary Figure S3A) to test both angiogenesis and endothelial stabilization. *HAS2* gene knockdown *in vitro* also resulted in increased angiogenesis in both the EC-pericyte coculture angiogenesis plexus assay (Figure 4A–C) and the Matrigel angiogenesis assay (Supplementary Figure S3B,C). Loss of HA expression in ECs cocultured with pericytes decreased the VE-cadherin (CD144) cell-cell binding complex (Figure 4D,F). Moreover, the tyrosine-protein kinase receptor TIE2 signaling (protein ratio p-TIE2 per TIE2) was decreased after knockdown of *HAS2* (Figure 4G,H). In contrast ICAM1 expression (mRNA) increased (Figure 4I) which indicates possible endothelial cell activation.

### 3.5. Endothelial HA Is Decreased in Diabetic Ischemic Muscle

**Figure 5:**
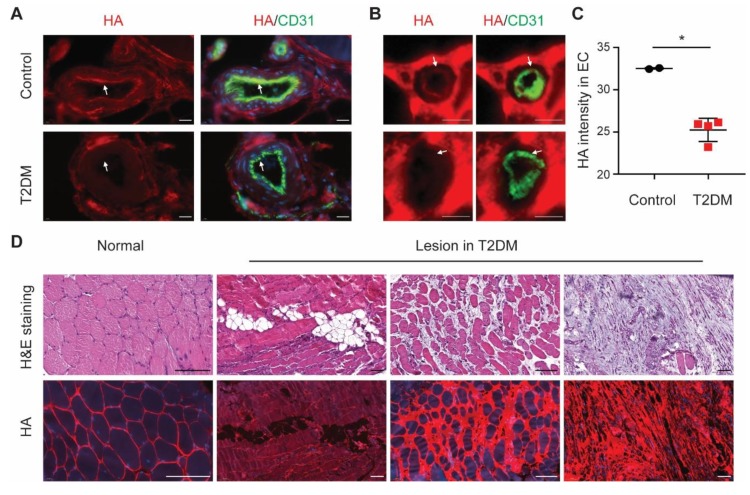
EC hyaluronan decreases in diabetic ischemic muscle. (**A**) Representative images of arteries (scale bar = 20 µm) and (**B**) small capillaries (scale bar = 5 µm) in ischemic muscle of type 2 diabetic patients with critical limb ischemia (*n* = 4) and control (*n* = 2), stained with the HA binding probe Ncan-dsRed (red) and CD31 (green) for endothelial localization. (**C**) Quantification of HA intensity colocalized with CD31 in ECs. (**D**) Representative images of H&E staining (top row) and HA staining (bottom row) in the normal and lesion area of ischemic muscle of type 2 diabetic foot patients (*n* = 4, scale bar = 100 µm). Values are given as mean ± SD. * *p* < 0.05.

Although the success of angiogenic therapy has been reported in young, healthy animals after acute ligation, these therapies so far failed to demonstrate clear therapeutic efficacy in diabetes patients with critical limb ischemia. Given the observations of increased HA shedding in diabetes [17,18], we hypothesized that in diabetes reduced endothelial HA expression causes endothelial dysfunction and, in turn, may attenuate the efficiency of angiogenic therapy. Muscle sections of critical limb ischemia biopsies of type 2 diabetic patients showed indeed a lower endothelial HA expression co-localizing with CD31 in blood vessels (Figure 5A–C) and, moreover, we also found increased adipocytes, edema, and myocyte degeneration (Figure 5D), similar to our observations in muscle tissue in *Has2*-cKO after ligation (Supplementary Figure S4). Non-endothelial ectopic accumulation of HA in the lesion area of the type 2 diabetic muscle could be observed (Figure 5C and Supplementary Figure S3; *Has2*-cKO).

## 4. Discussion

Our current study demonstrates a critical role for endothelial HA in microvascular perfusion and the angiogenic response to ischemic insults, such as can be found in type 2 diabetes patients. At a cellular level, loss of endothelial HA reduced Ang1/TIE2 signaling efficiency, which leads to vascular destabilization preventing complete microvascular reperfusion. In our, non-diabetic *Has2*-cKO mouse model, reduced endothelial HA after femoral artery ligation resulted in muscle damage, adipocyte presence, edema, and increased unstable capillaries which resembles the pathophysiology found in type 2 diabetic patient critical limb ischemia biopsies. 

HA plays a fundamental role in the maintenance of vascular integrity [20,25]. Removal of HA by hyaluronidase results in myocardial edema in perfused rat hearts [26]. Degradation of endothelial glycocalyx including HA is associated with reduced microvascular perfusion due to endothelial dysfunction and vascular injury [14,15]. High molecular weight HA enhances human pulmonary EC barrier function and inhibits LPS-induced pulmonary vascular leakiness [27]. Our recent study showed that endothelial specific deletion of has2 in mice leads to loss of HA incorporation in glycocalyx, causing endothelial dysfunction and vascular destabilization in multiple tissues, such as kidney, retina and heart [10]. Here, we further explored that loss of endothelial HA impaired microvascular perfusion and dysregulated angiogenesis in response to artery occlusion, which is clinically related to diabetic patients with critical limb ischemia.

An earlier study by our group showed that removal of endothelial HA using administration of the hyaluronan synthase inhibitor 4-methylesculetin (4ME) reduced the blood recovery after a double ligation surgery on both femoral and iliac arteries [19]. Loss of endothelial HA greatly impaired the collateral artery remodeling process in adductor muscle, due to the perturbed mechanotransduction [19]. Here we show that endothelial specific removal of HA induces vessel leakage and tissue edema in adductor muscle after ligation, suggesting inefficient vessel maturation and stabilization during remodeling. Moreover, this was associated with disturbed angiogenesis and impaired microcirculatory adaptation to ischemia. In this respect, it is of interest that addition of HA can enhance endothelial angiogenic cell therapy in the model of hindlimb ischemia [28]. In addition, biosynthesis of HA which is regulated by shear through the transcription factor Krüppel-like Factor 2 (KLF2), directly via increasing HAS2 protein expression and translocation of the enzyme to the cell membrane, and modulating endothelial glycolysis rate, through its activator 6-Phosphofructo-2-Kinase/Fructose-2,6-Biphosphatase 3 (PFKFB3) enzyme, influencing the rate limiting HA substrates UDP-glucosamine and UDP-glucuronic acid [29]. Thus, impaired perfusion can aggravate the process through reduced HA biosynthesis also.

These observations are particularly relevant to patients with diabetes, where we can demonstrate similar loss of HA from the microcirculation in critical limb ischemia. Diabetes is one of the major risk factors of atherosclerosis, as well as peripheral artery disease [30]. Critical limb ischemia is the most common cause of nontraumatic amputation in diabetes. In diabetes, angiogenic therapies such as gene therapy of growth factors [3,31] and bone marrow [32] have failed to achieve clear improvements.

This warrants a further understanding of how loss of HA from the glycocalyx leads to vessel destabilization. Angiogenesis is a process of capillary vessel growth from pre-existing vessels. Angiopoietin (Ang)/TIE2 signaling has been proposed as a critical factor to control sprouting angiogenesis, vascular remodeling and the transition between the quiescent and the activated phenotype of EC [33,34]. Decreased Ang1/TIE2 signaling by Ang2 upon VEGF or hypoxia, results in pericytes dissociation from EC and tip cells sprouting [34]. Ang1/TIE2 signaling also plays a very important role in vascular stability and integrity. Pericytes not only secrete growth factors, such as PDGF, VEGF, and Ang1, to promote the survival of endothelial cells but also keep the stability of vasculature through Ang1/TIE2 signaling. During neovascularization following ischemia, an initial decrease in TIE2 singling is essential for early vessel destabilization, while later on Ang1 is important for subsequent vessel maturation and functional neovascularization [35,36]. Our recent study shows that loss of endothelial HA greatly impaired the interaction with pericytes resulting in reduced Ang1/TIE2 signaling, which is accompanied with vascular leakage and disturbed angiogenesis. Interestingly, in a diabetes model Ang1 could promote stable neovascularization in a subcutaneous Matrigel [37].

In summary, endothelial HA plays a critical role in vessel stability which may be lost in diabetic limb ischemia. Our data point to targeting the balance between endothelial HA synthesis and degradation to restore Ang1/TIE2 signaling as a prerequisite to allow for microvascular adaptation to ischemia. This may include inhibiting hyaluronidases or increasing endothelial HA synthesis by modulating glycobiosynthesis.

## Supplementary Materials

The following are available online at https://www.mdpi.com/2073-4409/9/4/824/s1, Figure S1: Blood reperfusion measurement after hindlimb ligation surgery, Figure S2: H&E staining in regenerative region of calf muscle, Figure S3: Loss of hyaluronan in ECs increases angiogenesis *in vitro*, Figure S4: HA staining in regenerative region of calf muscle Table S1: Primers used for RT-PCR.
