# Supplementary material for: Loss of Endothelial Glycocalyx Hyaluronan Impairs Endothelial Stability and Adaptive Vascular Remodeling after Arterial Ischemia
Source: Cells. 2020 Mar 29;9(4):824. doi: 10.3390/cells9040824 (PMC7226746; doi:10.3390/cells9040824)
Supplement: Supplementary file 1 [file cells-09-00824-s001.pdf]

Loss of endothelial glycocalyx hyaluronan impairs endothelial stability and adaptive vascular remodeling after arterial ischemia

Gangqi Wang[1], Margreet R. de Vries[2], Wendy M.P.J. Sol[1], Annemarie M. van Oeveren-Rietdijk[1], Hetty C. de Boer[1], Anton Jan van Zonneveld[1], Paul H.A. Quax[2], Ton J. Rabelink[1] and Bernard M. van den Berg[1*]

1 The Einthoven laboratory for Vascular and Regenerative Medicine, Department of Internal Medicine, Division of Nephrology, Leiden University Medical Center, Leiden, the Netherlands.

2 The Einthoven laboratory for Vascular and Regenerative Medicine, Department of Surgery, Leiden University Medical Center, Leiden, the Netherlands.

* Corresponding : Email: bmvandenberg@lumc.nl; Tel: +31 71 52 65024; Leiden University Medical Center, Albinusdreef 2, 2333 ZA, Leiden, the Netherlands

Supplemental Methods

Mice and Experimental groups

Endothelial specific conditional homozygous has2 knockout (*Has2*-cKO) mice were generated as descripted in our previous work [1]. Animal experiments were approved by the ethical committee on animal care and experimentation of the Leiden University Medical Center (permit no. 14-150). All animal work was performed in compliance with the Dutch government guidelines and the Directive 2010/63/EU of the European Parliament. Presence of introduced genes from ear pinched DNA samples was performed on all animals during breeding and for confirmation of *has2* gene mutation (exon 2 deletion) after tamoxifen administration on *Has2*-cKO mice at the end of the experiment using PCR and primers [1]. Only *Has2*-cKO mice with a confirmed mutation within their *has2* gene were examined further. Eight-week-old male *Has2*-cKO and control mice received 2 mg/0.2 mL tamoxifen intra-peritoneally for 5 consecutive days. 4 weeks after tamoxifen, a single ligation surgery or a double ligation surgery was performed in both *Has2*-cKO and control mice, followed with Laser Doppler Perfusion Imaging (LDPI) and body weight measurement for 14 days. The blood was taken both one day before ligation and on the day of sacrifice. Under Isoflurane anesthesia, mice was sacrificed by heart punction for blood collection and perfusion fixation. Both adductor muscle and calf muscle were collected and embedded in paraffin.

Hindlimb ligation surgery and Laser Doppler perfusion Imaging (LDPI)

The details of double and single ligation surgery were described in the previous study [2]. In brief, for a double ligation model, both common iliac artery and femoral artery were electrocoagulated, and for single ligation model, the left common femoral artery proximal to the bifurcation of the popliteal and saphenous artery was electrocoagulated. Before surgery mice were anaesthetised with an intra-peritoneal injection of midazolam (5 mg kg^{-1}, Roche), medetomidine (0.5 mg kg^{-1}, Orion) and fentanyl (0.08 mg/kg, Janssen Pharmaceuticals). After surgery, anaesthesia was antagonised with flumazenil (0.7 mg/kg, Fresenius Kabi) and buprenorphine (0.2 mg/kg, MSD Animal Health) was administered as analgesic. Before LDPI mice were anaesthetised with an intra-peritoneal injection of midazolam and medetomidine and mice were kept in a double-glassed jar filled with a 37°C water mantle during the whole measurement. Before and after ligation, perfusion was measured using Laser Doppler Perfusion Imaging (Moor Instruments). The perfusion values were analyzed using Moor LDI V5.3 software.

Human tissue samples

Human tissue samples from patients with critical limb ischemia and type 2 diabetes were collected at the Leiden University Medical Center. This study was conducted according to the Declaration of Helsinki. Sample collection was approved by the Medical Ethics Committee of the Leiden University Medical Center (Protocol No. P12.265) and written informed consent was obtained from the participants.

Inclusion criteria were a minimum age of 18 years and lower limb amputation, excluding ankle, foot, or toe amputations. The exclusion criteria were suspected or confirmed malignancy and inability to give informed consent. Tissue samples were obtained directly after lower limb amputation, snap-frozen and stored at -80°C.

Histology

Deparaffinized muscle sections (4 µm thick) were rinsed in Millipore water and stained with Mayers Haematoxylin (109249, Merck) for 1 min. Slides were rinsed in tap water and kept on a orbitalshaker for 10 min for further rinsing with milliQ water. Next, slides were incubated with an ethanol-based eosin (E6003, Sigma-Aldrich) solution for 2 min. Slides were dehydrated in absolute ethanol and xylene and mounted.

Immunohistochemistry

Deparaffinized muscle sections (4 µm thick) were washed in PBS and antigen retrieval was performed using antigen retrieval buffer (Dako) in an autoclave. Slides were blocked in Serum-Free Protein Block buffer (Dako) for 1 hour at room temperature. Primary Goat anti-mouse Ang1 (AF923, R&D Systems), rabbit anti laminin (Z0097, Dako), rat anti-mouse pdgfrβ (ab51876, Abcam), HA binding peptide Neurocan-dsRed [1] (200 µg/mL), or lectin from *Bandeiraea simplicifolia* isolectin B4 (BS-1-TRITC, L5264, Sigma or BS-1-BIOTIN, L2140, Sigma) were incubated overnight at 4°C in blocking buffer, followed by an appropriate secondary antibodies for 1 hour at room temperature. Human diabetic muscle sections (4 µm thick) of critical limb ischemia biopsies treated as above and incubated overnight with Ncan-dsRed (200 µg/mL) and Rabbit anti-Human CD31 antibody (ab28364, Abcam) at 4°C, followed by an appropriate secondary antibody for 1 hour in blocking buffer, washed with PBS.

Tissue slides were embedded in Prolong^{TM} gold antifade mountant with DAPI (P36931, ThermoFisher) and recorded using a 3D Histech Pannoramic MIDI Scanner (Sysmex). Quantification was performed using the public domain NIH ImageJ software (FIJI version 1.49m; http://rsb.info.nih.gov/ij).

Viral transduction

The hyaluronan synthase 2-short-hairpin RNA lentivirus (pLV-*CMV-IE.HAS2shRNA*) was created through transfecting Hek293 cells (60-80% confluence) with a mixture of combined plasmid DNA of *HAS2shRNA* (Sigma-Aldrich, Mission shRNA library TRCN0000045394), H1/VSVG and H23/pspax2 with polyethylenimine (PEI, 0.05 mg/mL final concentration) in DMEM (Gibco) to obtain the self-inactivating (SIN) lentiviral construct from supernatant. Primary human glomerular-derived microvascular endothelial cells (hMVECs) or primary Human umbilical vein endothelial cells (HUVECs) were cultured to 60-80% confluency in T75 flasks (Greiner bio-one, Alphen a/d Rijn, the Netherlands) in EGM2 (Lonza) medium and transduced with pLV-CMV-IE.HAS2shRNA or mock (pLV-CMV-IE) in combination with 8 µg/mL polybrene, incubated overnight at 37°C and 5% CO_2. After medium refreshment experimental assays were started.

Angiogenesis plexus assay

Kidney mesenchymal stromal cells (kMSCs, 2 x 10^4 cells) and hMVECs (1 x 10^4 cells) were mixed and seeded in 1% gelatin coated 96 well plate, cultured in EC-SFM medium (Gibco) with 1% platelet poor plasma derived serum, 30 ng/mL VEGF-165 (R&D) and 20 ng/ mL bFGF (Miltenyi). Pellet cells together upon centrifugation for 30 seconds at 300 x g. The cells were cultured for 7 days until vascular tubes formed. Next, cells were fixed with 4% PFA and 0.2% Triton-X100 in PBS for 10 min at room temperature, washed with PBS, and blocked for 1 hour at room temperature in 5% BSA in PBS. Primary mouse anti-human CD31 (555445, BD Biosciences) and a-SMA (C6198, Sigma) were incubated overnight at 4°C, followed by an appropriate secondary antibodies and Hoechst 33528 for 1 hour, all in blocking buffer. Cells were examined using a LEICA TCS SP8 X WLL (Leica, Rijswijk, The Netherlands) and a 60x objective (HC PL APO CS2 40x/1.30 OIL, Leica). Sequential 16-bit confocal images (xyz dimensions, 0.142×0.142×0.3µm) were recorded using LAS-X Image software (Leica) and analysed with ImageJ. Both vascular area and vascular branches were quantified.

Matrigel angiogenesis assay

96 Well-plates were coated with 46µL of growth factor reduced Matrigel (354230, Corning) and incubated at 37°C for 1 hour to let the gel solidify. HUVECs were seeded at a density of 20000/well and images were taken every 15 min using Leica AF6000 microscope at 37°C and 5% CO_2 for up to 5hrs. Total branch length and tube number at 5hrs were quantified using ImageJ software.

EC/pericyte coculture assay

Kidney mesenchymal stromal cells (kMSCs, 2 x 10^4 cells) were seeded in 1% gelatin coated 96 well plate. After 4 hours when all the cells adherent to the plate, HUVECs (2 x 10^4 cells) were added on top of the kMSCs, cultured in EGM2 (Lonza) medium. The cells were cultured for 2 days until a confluent endothelial monolayer formed. Next, cells were fixed with 4% PFA and 0.2% Triton-X100 in PBS for 10 min at room temperature, washed with PBS, and blocked for 1 hour at room temperature in 3% normal goat serum and 2% BSA in PBS. Primary monoclonal Mouse Anti-Human VE cadherin (CD144, 55-7H1, BD Biosciences) and phalloidin-TRITC (P1951, Sigma) were incubated overnight at 4°C, followed by an appropriate secondary antibody and Hoechst 33528 for 1 hour, all in blocking buffer. Cells were examined using a LEICA TCS SP8 X WLL (Leica, Rijswijk, The Netherlands) and a 60x objective (HC PL APO CS2 40x/1.30 OIL, Leica). Sequential 16-bit confocal images (xyz dimensions, 0.142×0.142×0.3µm) were recorded using LAS-X Image software (Leica) and analysed with ImageJ. The stable linear adherence junctions was quantified as ratio over total junction length.

Real-time PCR

Cells were harvested in Trizol reagent (15596018, Life Technologies). Total RNA was isolated using RNeasy mini kit (74106, Qiagen) according to its protocol. cDNA was synthesized by mixing 1 mg total RNA, Oligo (dT) (C110A, Promega) and dNTP (C110A, Promega) and incubate at 65 ℃ for 5 min firstly. Then M-MLV reverse transcriptase (M170B, Promega), recombinant RNasin® ribonuclease inhibitor (N251B, Promega) and DTT were added into the mixture and incubate at 37 ℃ for 50 min. terminate the reaction by incubating at 70 ℃ for 15 min. SYBR select master mix (4472897, Applied Biosystems™) and specific primers (Table S1) was used for real time PCR. The expression of genes were determined by normalized to GAPDH levels.

Immunoblotting

Western blots were performed form protein extracts of HUVECS as described in supplemental materials. Cells were washed with PBS and lysed in 50mM TRIS HCL buffer (pH 7.5) containing 150mM NaCl, 1% SDS, 0.5% deoxycholate and 0.5% triton X-100. Protein samples were diluted 5x in SDS sample buffer (pH 6.8; 10% SDS, 25% 2-mercaptoethanol, 50% glycerol, 0.01% bromophenol blue, 0.3125M Tris-HCl, 0.5M DTT), incubated at 95°C for 10 minutes and subjected to SDS-PAGE and western blotting. Proteins, transferred to PVDF membrane (1704156, Bio-Rad Laboratories BV, Veenendaal, The Netherlands) were detected with antibodies against Phospho-Tie2 (Y992; AF2720-SP, R&D Systems), Pospho-Tie2 (Y1102/Y1100; AF3909, R&D Systems), and Tie2 (557039, BD Biosciences), after blocking the membrane with 5% fat-free milk in PBS and 0.1% Tween-20. Followed by incubation with a secondary HRP-conjugated antibody and Pierce ECL Western Blotting substrate (32106, Thermo Scientific). Band intensity was analysed using ImageJ software.

Statistical analysis

Data are presented as mean ± SD, unless indicated otherwise. For all experiments, 3-5 biological replicates were performed. Differences between groups were assessed by non-paired 2-tailed Student's t test, paired 2-tailed Student's t test or, when not normally distributed, by two-tailed F-test. P values < 0.05 were considered statistically significant.

Supplemental figures

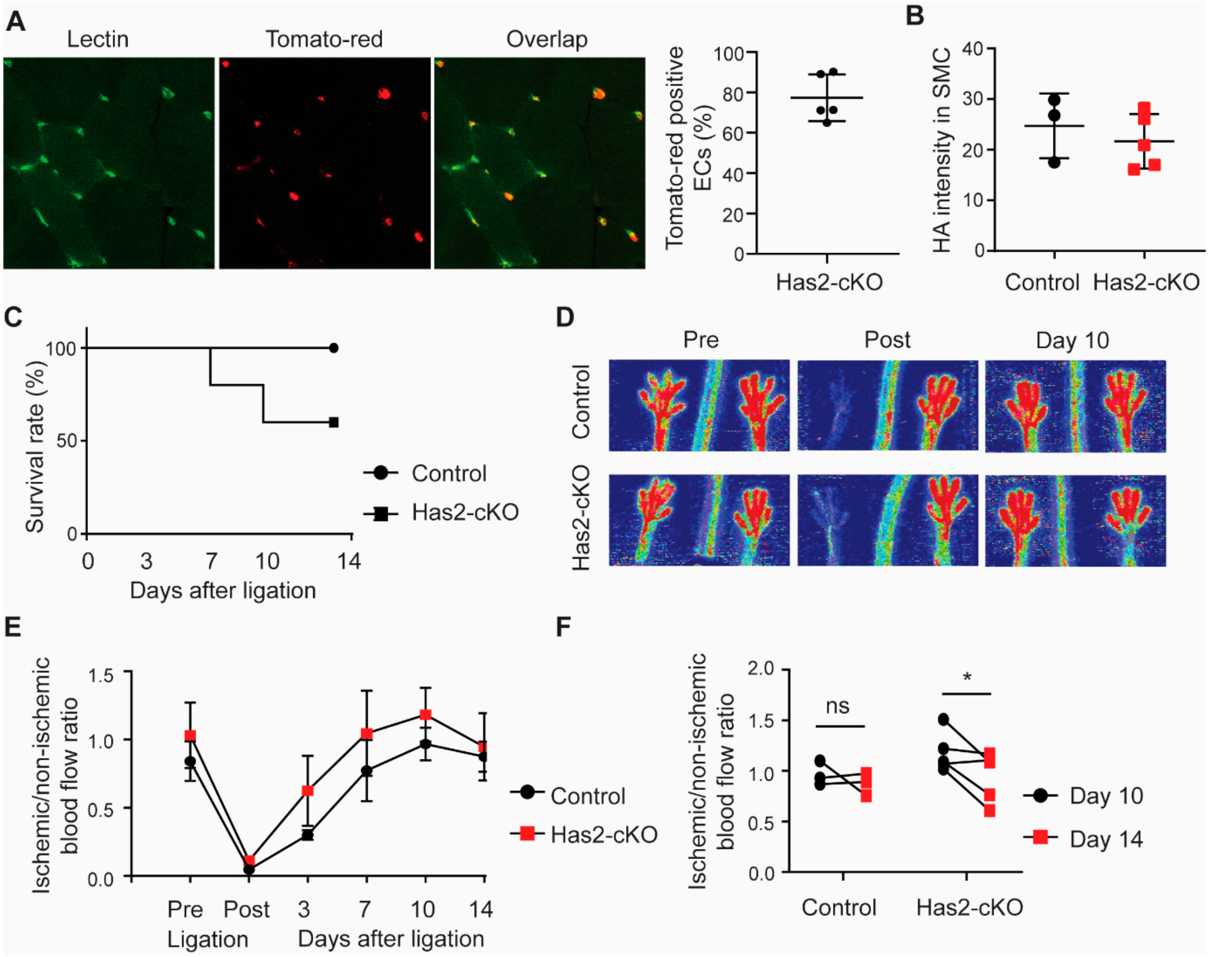

Figure S1: Blood reperfusion measurement after hindlimb ligation surgery.

(**A**) Representative images of BS-I endothelial staining (lectin, green) and tomato-red reporter staining (red) in calf muscle tissue of Has2-cKO mice after tamoxifen induction and quantification of efficiency by percentage tomato-red positive endothelial cells (n=5). (**B**) Quantification of HA intensity colocalized with α-SMA in smooth muscle cells. (**C**) Survival graph of Has2-cKO (n=5) and control (n=5) mice after double coagulation of femoral and femoral artery. (**D**) Representative images of paw perfusion by laser Doppler perfusion imaging (LDPI) after single coagulation of femoral artery in Has2-cKO (n=5) and control (n=3) mice. (**E**), (**F**) Quantification of blood reperfusion ratio after single ligation of femoral artery in hindlimb of Has2-cKO (n=5) and control (n=3) mice. Values are given as mean ± SD. Paired 1-tailed Student's t test was performed; *P < 0.05.

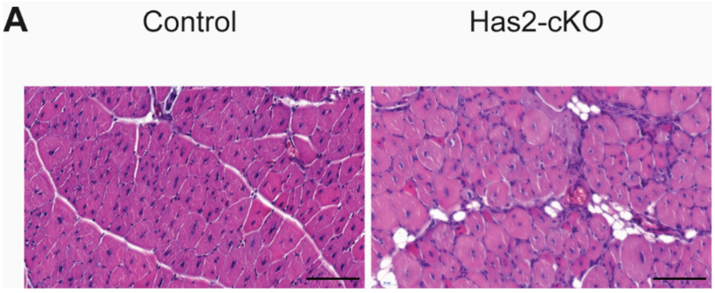

Figure S2: H&E staining in regenerative region of calf muscle.

(**A**) Representative images of H&E staining show adipocytes infiltration in Has2-cKO mice (n=5) after single femoral artery ligation (scale bar = 100 µm).

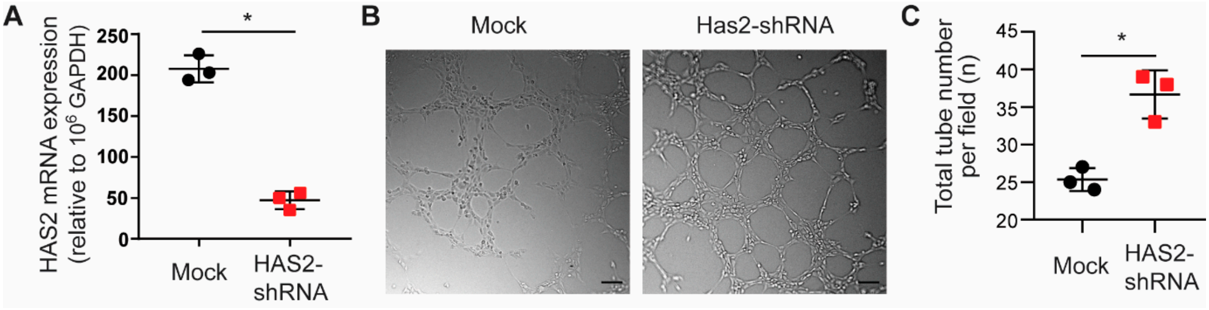

Figure S3: Loss of hyaluronan in ECs increases angiogenesis *in vitro*.

(**A**) HAS2 mRNA expression after short hairpin RNA (*HAS2*-shRNA) treatment. (**B**) Representative images of Matrigel angiogenesis assay with silencing of Has2 (scale bar = 100 µm). (**C**) Quantification of tube number show increased angiogenesis with silencing of Has2 (n=3). Values are given as mean ± SD. Non-paired 2-tailed Student's t test was performed; *P < 0.05.

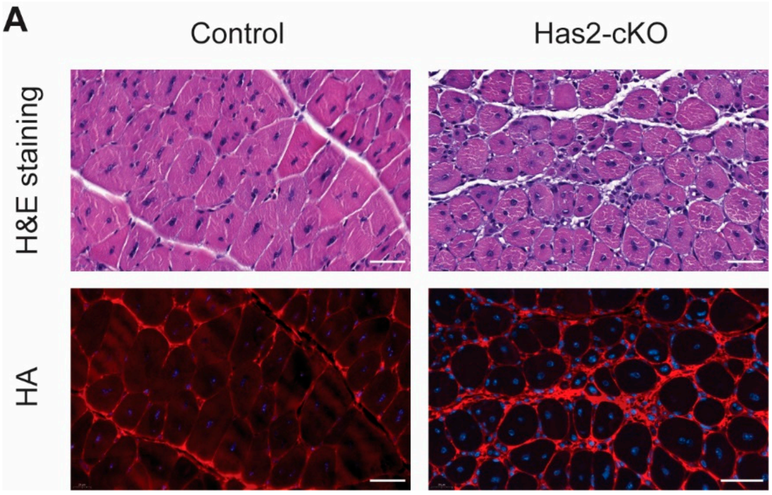

Figure S4: HA staining in regenerative region of calf muscle.

(**A**) Representative images of H&E staining (top row) and HA staining (bottom row) in the regenerative region of calf muscle from both Has2-Cko (n=5) and control (n=3) mice (scale bar = 40 µm).

Table S1 Primers used for RT-PCR

	Forward (5'-3')	Reverse (5'-3')	Product (bp)
ICAM1	GTATGAACTGAGCAATGTGCAAG	GTTCCACCCGTTCTGGAGTC	119
HAS2	AAGAACAACTTCCACGAAAAGGG	GGCTGGGTCAAGCATAGTGT	216
GAPDH	TTCCAGGAGCGAGATCCCT	CACCCATGACGAACATGGG	175

3. References

1. van den Berg, B.M.; Wang, G.; Boels, M.G.S.; Avramut, M.C.; Jansen, E.; Sol, W.; Lebrin, F.; Jan van Zonneveld, A.; de Koning, E.J.P.; Vink, H., et al. Glomerular Function and Structural Integrity Depend on Hyaluronan Synthesis by Glomerular Endothelium. *J Am Soc Nephrol* **2019**, 10.1681/ASN.2019020192, DOI: 10.1681/ASN.2019020192, doi:10.1681/ASN.2019020192.
2. Hellingman, A.A.; Bastiaansen, A.J.; de Vries, M.R.; Seghers, L.; Lijkwan, M.A.; Lowik, C.W.; Hamming, J.F.; Quax, P.H. Variations in surgical procedures for hind limb ischaemia mouse models result in differences in collateral formation. *European journal of vascular and endovascular surgery : the official journal of the European Society for Vascular Surgery* **2010**, *40*, 796-803, doi:10.1016/j.ejvs.2010.07.009.
